# With Over 60 Independent Losses, Stomata Are Expendable in Mosses

**DOI:** 10.3389/fpls.2020.00567

**Published:** 2020-05-28

**Authors:** Karen S. Renzaglia, William B. Browning, Amelia Merced

**Affiliations:** ^1^Plant Biology Department, Southern Illinois University, Carbondale, IL, United States; ^2^International Institute of Tropical Forestry, USDA Forest Service, San Juan, PR, United States

**Keywords:** stomata, mosses, guard cells, intercellular space, capsule, land plant evolution

## Abstract

Because stomata in bryophytes are uniquely located on sporangia, the physiological and evolutionary constraints placed on bryophyte stomata are fundamentally different from those on leaves of tracheophytes. Although losses of stomata have been documented in mosses, the extent to which this evolutionary process occurred remains relatively unexplored. We initiated this study by plotting the known occurrences of stomata loss and numbers per capsule on the most recent moss phylogeny. From this, we identified 40 families and 74 genera that lack stomata, of which at least 63 are independent losses. No trends in stomata losses or numbers are evident in any direction across moss diversity. Extant taxa in early divergent moss lineages either lack stomata or produce pseudostomata that do not form pores. The earliest land plant macrofossils from 400 ma exhibit similar sporangial morphologies and stomatal distribution to extant mosses, suggesting that the earliest mosses may have possessed and lost stomata as is common in the group. To understand why stomata are expendable in mosses, we conducted comparative anatomical studies on a range of mosses with and without stomata. We compared the anatomy of stomate and astomate taxa and the development of intercellular spaces, including substomatal cavities, across mosses. Two types of intercellular spaces that develop differently are seen in peristomate mosses, those associated with stomata and those that surround the spore sac. Capsule architecture in astomate mosses ranges from solid in the taxa in early divergent lineages to containing an internal space that is directly connected to the conducing tissue and is involved in capsule expansion and the nourishment, hydration and development of spores. This anatomy reveals there are different architectural arrangements of tissues within moss capsules that are equally effective in accomplishing the essential processes of sporogenesis and spore dispersal. Stomata are not foundational to these processes.

## Introduction

Stomata in bryophytes are located on sporangia and are restricted in their occurrence across phylogeny. Liverworts are the only extant land plants that lack stomata entirely, while stomata are widespread but not ubiquitous in hornworts and mosses. With contemporary phylogenies pointing to hornworts as the earliest divergent bryophyte group (Puttick et al., 2018; Renzaglia et al., 2018), stomata are best interpreted as plesiomorphic in land plants, especially given that *Leiosporoceros*, the sister taxon to other hornworts, possesses stomata. Within the small hornwort clade of 10–12 genera there are two well-documented losses of stomata in derived taxa (Renzaglia et al., 2017). In comparison, early diversification of the moss assemblage apparently was not dependent on the existence of stomata as Takakiales and Andreaeopsida, two of the oldest moss clades, are stomata free. Moreover, there are multiple moss orders and families that include taxa with and without stomata. Clearly stomata are not vital to the survival and were not required for the initial radiation of bryophytes.

The sporadic occurrence of stomata in bryophytes calls into question the role stomata play in the physiology and growth of bryophyte sporophytes. Recent studies reveal that diurnal cycles of opening and closing, and responses to ABA and desiccation, which are key to water relations in tracheophytes, do not occur in hornworts (Pressel et al., 2018). However, substomatal cavities and intercellular spaces that are necessary for functional stomata are always present in mosses and hornworts with stomata, while species without stomata do not have substomatal spaces (Goffinet et al., 2009; Merced and Renzaglia, 2017). Intercellular spaces are common in different tissues of land plants, and in some bryophytes are present in both gametophyte and sporophyte generations, suggesting that spaces originated multiple times in the evolution of plants (Duckett and Pressel, 2018). In tracheophytes, intercellular spaces in the form of spongy tissue are coordinated with the presence of functional stomata to facilitate gas exchange (Dow et al., 2017; Lundgren et al., 2019).

In order to better understand the evolution of stomata within mosses, we traced the number per capsule, and known absence of stomata across the range of moss diversity. Due to the lack of stomata in early divergent moss lineages, we examined the fossil record on early land plants for clues to the origin of the moss capsule with and without stomata. We tested the hypothesis that stomata were lost repeatedly throughout the history of mosses and not restricted to derived taxa. We further speculated that stomatal losses were accompanied by anatomical and developmental modification within the sporophyte. Accordingly, we identified architectural features that characterize sporophytes with and without stomata and documented the development of intercellular spaces, including substomatal cavities. Anatomical and developmental analyses identify two distinct types of internal spaces in mosses and document the loss of peripheral spaces strictly associated with guard cells and the retention of internal spaces in taxa without stomata. Our anatomical studies point to modified architectural features that accompanied stomata loss and led to fundamentally different, but equally effective, internal hydration and capsule maturation.

## Materials and Methods

### Plants and Specimens Examined

Moss capsules were collected locally in Southern Illinois over the growing season to ensure observations of early and late stages of development. Prepared blocks of capsules from species not found in Illinois were sectioned and examined. Species examined include the following, with the seven taxa lacking stomata denoted by asterisks: *Takakia ceratophylla*^∗^, *Andreaea rothii*^∗^, *Sphagnum angustifolium*^∗^, *Polytrichastrum ohiensis*, *Atrichum angustatum*^∗^, *Tetraphis pellucida*^∗^, *Diphysium foliosum*, *Buxbaumia viridis*, *Physcomitrium pomiform*, *Physcomitrium* (*Physcomitrella) patens*, *Funaria hygrometrica*, *Dicranum scoparium*, *Orthotrichum pusillum*, *Plagiomnium cuspidatum*, *Ephemerum spinosum*, *Leucobryum glaucum*^∗^, *Bartramia pomiforme*, *Hypnum curvifolium*, *Brachythecium rutabulum*, *Thuidium delicatulum*, and *Neckeropsis undulata*.^∗^ A KNOX mutant of *P. patens* that lacks stomata was acquired from Dr. Neil Ashton.

Published records of fossils of the earliest land plants with sporangia and stomata were examined for comparisons with the morphology and anatomy of the extant members of early divergent moss lineages. Fossil stomata were reproduced from Edwards (1979) and Edwards et al. (1998) with permission.

### Stomata Presence in the Phylogeny of Mosses

**FIGURE 1:**
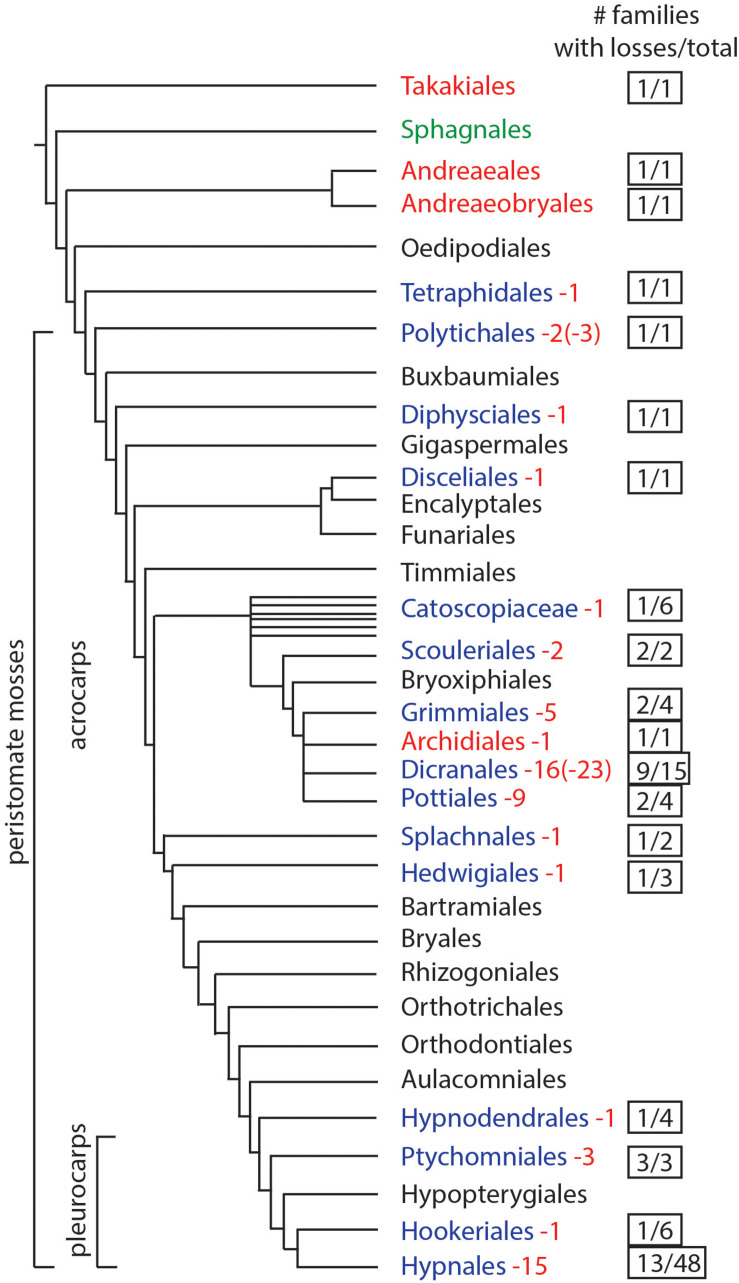
Phylogenetic of moss orders based on Liu et al. (2019). Orders in red lack stomata, green have pseudostomata, black have stomata (no records of losses), and blue have documented losses of stomata. Numbers in red represent the minimum times stomata were lost and numbers in parentheses indicate the maximum possible number of losses. The right column indicates the number of families that include taxa without stomata, over the total number of families in the order.

We assessed the presence and absence of stomata by mapping their occurrence across the most recent phylogeny of mosses (Liu et al., 2019). An extensive literature review (Table 1) identified genera and species that lack stomata, and confirmed the number of stomata reported for members of each moss family, if known. To determine the minimum number of losses in moss orders, we counted the number of families that have genera that lack stomata and assessed independent origin based on phylogenetic relationships. If a genus has species with both states (present and absent stomata) these were counted as independent losses. Losses within different families were each scored as independent. From these analyses, we estimate the minimum number and, in some cases, maximum number (in parenthesis) of losses for each order (Figure 1).

**TABLE 1:** Counts per capsule and 40 losses (counts of 0) of stomata in 69 families of mosses.

Family	Stomata per capsule	References (in Supplementary Material)
Oedipodiaceae	60	Paton and Pearce, 1957
**Polytichaceae**	0, 20, 40, 50–78, 80–***120, 200, 250***	Paton and Pearce, 1957; Duckett and Pressel, 2018
Tetraphidaceae	0, 5	Paton and Pearce, 1957
Buxbaumiaceae	20–30	Paton and Pearce, 1957, present study
**Diphysciaceae**	0, 10	Paton and Pearce, 1957; Schofield, 2007
Timmiaceae	30	Paton and Pearce, 1957
**Disceliaceae**	0, ?	Paton and Pearce, 1957
Encalyptaceae	15, 30, 50	Paton and Pearce, 1957
Funariaceae	10, 14, 60, *160, 180, 200*	Paton and Pearce, 1957; Duckett and Pressel, 2018
Catoscopiaceae	0, ?	Paton and Pearce, 1957
Distichiaceae	8–12	Paton and Pearce, 1957
**Scouleriaceae**	0*	Duckett and Pressel, 2018
Drummundiaceae	0, ?	Vitt, 2007
Saelaniaceae	6	Paton and Pearce, 1957
**Grimmiacea**	0, 6–18, 30	Paton and Pearce, 1957; Hastings and Grevens, 2007; McIntosh, 2007
**Seligeriaceae**	0, 4, 8	Paton and Pearce, 1957; Bartlett and Vitt, 1986; Andreas, 2013; Duckett and Pressel, 2018
**Archidiaceae**	0*	Paton and Pearce, 1957
Fissidentaceae	0, 12	Paton and Pearce, 1957; Egunyomi, 1982; Pursell, 1987, 2007; Pursell et al., 1988
**Ditrichaceae**	0, 4, 6, 8–12	Paton and Pearce, 1957; Gradstein et al., 2001
Bruchiaceae	0, 70	Tong and He, 2002
Erpodiaceae	0, ?	Gradstein et al., 2001; Milne and Klazenga, 2012
Schistostegaceae	0, 4, 5	Jennings, 1913; Paton and Pearce, 1957
Rhabdoweisiaceae	5–12	Paton and Pearce, 1957
Dicranaceae	0, 4, 6–20	Paton and Pearce, 1957; Gradstein et al., 2001; Duckett and Pressel, 2018
Micromitriaceae	0, ?	Crum and Anderson, 1981; Smith, 2004; Bryan, 2007
**Leucobryaceae**	0, ?	Paton and Pearce, 1957; Gradstein et al., 2001
Calymperaceae	0, 2, 15	Egunyomi, 1982
**Pottiaceae**	0, 3–16	Paton and Pearce, 1957; Egunyomi, 1982; Zander and Eckel, 1993; Abella et al., 1999; Gradstein et al., 2001
Pleurophascaceae	0, ?	Fife and Dalton, 2005
**Splachnaceae**	20, 30, 40, 50, 60, 90	Paton and Pearce, 1957; Goffinet, 2012
Meesiaceae	30, 50, 70	Paton and Pearce, 1957
Bryaceae	15, 50–70, 90, *100, 120, 185*	Paton and Pearce, 1957; Egunyomi, 1982; Duckett and Pressel, 2018, present study
Mniaceae	8–20, 40, 45, 60, *180*	Paton and Pearce, 1957, present study
Bartramiaceae	16, 28, 40, 45, 60, 70, ***100, 220–240***	Paton and Pearce, 1957
Orthotrichaceae	3–8, 12, 20, 40	Paton and Pearce, 1957, present study
Hedwigiaceae	0, 12, 24	Paton and Pearce, 1957; Vitt and Buck, 1984
Aulacomniaceae	6–12, 30	Paton and Pearce, 1957
Orthodontiaceae	14, 18	Paton and Pearce, 1957
Pterobryellaceae	0, ?	Arzeni, 1954
Orthorrhynchiaceae	0, ?	Klazenga, 2012a
Rhabdodontiaceae	0, ?	Paton and Pearce, 1957; Hattaway, 1984
**Ptychomniceae**	0, 5, 10	Paton and Pearce, 1957; During, 1977; Hattaway, 1984
Daltoniaceae	10	Paton and Pearce, 1957; Gradstein et al., 2001
Hookeriaceae	18	Paton and Pearce, 1957
Pilotrichaceae	0, ?	Paton and Pearce, 1957
Fontinalaceae	0*	Paton and Pearce, 1957; Allen, 2015
Climaciaceae	12	Paton and Pearce, 1957
Amblystegiaceae	6–50, 54, 80, ***130, 200***	Paton and Pearce, 1957; Cheney, 1897
Helodiaceae	20	Paton and Pearce, 1957
Leskeaceae	0, 4–6, 8, 20	Buck, 1981; Spence, 2015
Thuidiaceae	5, 24	Paton and Pearce, 1957; Egunyomi, 1982
Stereophyllaceae	0, 6, 23	Egunyomi, 1982
Brachytheciaceae	5–28, 30	Paton and Pearce, 1957
Myriniaceae	14	Paton and Pearce, 1957; Gradstein et al., 2001
Fabroniaceae	4	Egunyomi, 1982
Hypnaceae	3, 4, 5–10, 11, 16, 19–44	Paton and Pearce, 1957; Egunyomi, 1982, present study
Pterigynandraceae	8, 10	Klazenga, 2012b
Hylocomiaceae	6–15, 22	Paton and Pearce, 1957; Hedenäs, 2005
Plagiotheciaceae	0, 5–10, 14, 20	Paton and Pearce, 1957; Ireland, 2015
Entodontaceae	0, 8	Paton and Pearce, 1957; Gradstein et al., 2001
Pylaisiadelphaceae	4, 6, 8	Paton and Pearce, 1957; Gradstein et al., 2001
Sematophyllaceae	0, 4	Paton and Pearce, 1957; Fife, 2012
Cryphaeaceae	0, 12	Paton and Pearce, 1957; Gradstein et al., 2001
Leucodontaceae	0, 12	Paton and Pearce, 1957
Pterobryaceae	0*	Yu and Jia, 2012
Neckeraceae	0, 4, 12, 14	Paton and Pearce, 1957; Gradstein et al., 2001
Leptodontaceae	0, 6	Paton and Pearce, 1957; Stark, 2015
Lembophyllaceae	0, 10–20, 90	Paton and Pearce, 1957; Tangney, 1997; Gradstein et al., 2001
Anomodontaceae	0, 12	Gradstein et al., 2001

For families with losses (counts of 0), “?” denotes no reports of counts in stomata-containing members and * denotes no stomata-containing members. Counts greater than 100 in six families are in bold and italicized.

### Light Microscopy, Electron Microscopy, and Immunogold Labeling

Protocols are described in detail in Merced and Renzaglia (2013, 2014). Sporophytes of mosses were fixed in 2% glutaraldehyde in 0.05M NaPO_4_ buffer, washed three times in 0.05M NaPO_4_ buffer and post-fixed for 20 min in 1% OsO4 in 0.05M NaPO_4_ buffer. Specimens were rinsed in distilled water and dehydrated in a graded ethanol series ending with 100% ethanol. For scanning electron microscopy (SEM), fixed capsules were critical point dried and mounted on stubs, then sputter-coated for 230 s with palladium-gold. Specimens were observed using a Hitachi S570 scanning electron microscope. For light microscopy and transmission electron microscopy (TEM), specimens were infiltrated with Spurr’s resin (Electron Microscopy Sciences, Hatfield, PA, United States) or LR White resin (London Resin Company, Berkshire, United Kingdom) and cured at 65°C. For light microscopy, semi-thin sections (250–750 nm) were mounted on glass slides and stained with 1.5% toluidine blue in distilled water. Slides were observed on a Leica DM5000 B compound microscope and images captured digitally. Thin sections (60–90 nm) were collected on nickel grids, incubated with 2% BSA in 0.02M PBS for immunogold labeling. Grids were transferred to the LM19 primary antibody (diluted 1: 20 in 2% BSA/PBS) for 3 h and controls (one grid each treatment) were left in buffer during that time. All grids were rinsed in 2% BSA/PBS, then incubated in goat anti-rat IgG secondary antibody (Sigma- Aldrich, St. Louis, MO, United States) diluted 1: 20 in 2% BSA/PBS for 30 min. Grids were rinsed with PBS followed by distilled/deionized autoclaved filtered water, and dried at room temperature. Grids were observed unstained with a Hitachi H7650 transmission electron microscope at 60 kV.

## Results

### Stomata Presence and Number in the Phylogeny of Mosses

**FIGURE 2:**
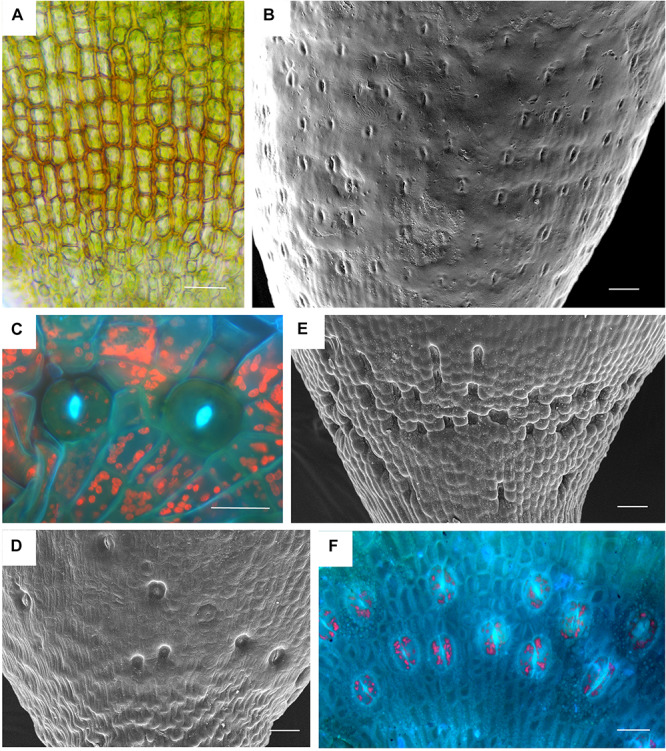
Stomata diversity in mosses. **(A)** *Atrichum angustatum* light micrograph of stomata free epidermis. **(B)** *Funaria hygrometrica* SEM of apophysis covered with ∼200 stomata. **(C)** *Physcomitrium* (*Physcomitrella) patens* 2 of 10 stomata in fluorescence. **(D)** *Brachythecium rutabulum* SEM of sparse scattered stomata. Image credit: Jeffrey J. Duckett. **(E)** *Plagiomnium cuspidatum* SEM showing numerous sunken stomata on the apophysis. 60 stomata estimated in the capsule. Image credit: Jeffrey J. Duckett. **(F)** *Bartramia pomiforme* group of stomata in fluorescence. 70 stomata estimated in the capsule. Bars: **(A,C,F)** = 20 μm, **(B,D,E)** = 50 μm.

Based on data mining from published literature, stomata are absent in 74 genera and 40 families of mosses, accounting for at least 63 independent losses in the phylogeny of mosses (Figure 1 and Supplementary Data). Nearly 60% (16 of 28) of the orders of peristomate mosses have recorded losses of stomata. These losses are equally present in acrocarps and pleurocarps with high numbers in the Dicranales, Pottiales, and Hypnales (Figure 1). As the sister taxon to peristomate mosses, *Oedipodium* represents the earliest divergent moss lineage to possess stomata. Numbers of stomata per capsule range from 0 to 250 (Figure 2 and Table 1), with the vast majority of counts (40 of 54 = 74%) ranging from 3 to 30 (Figures 2C,D). Numbers above 200 are rare and recorded only for three families, Polytrichaceae, Funariaceae (Figure 2B) and Bartramiaceae, although many members of these family have less than 70 stomata (Figures 2C,F) (Table 1). There are no evident trends in numbers in either direction with divergence time. For example, numbers vary in the first moss lineages with stomata: in *Oedipodium* the 60 or so stomata are scattered along the highly elongated neck and within the Tetraphidaceae, *Tetraphis* lacks stomata and *Tetradontium* contains only five per capsule. Members of the Polytrichales exhibit the extremes in stomata numbers per capsule, with 200 and 250 in *Polytrichum* and zero in three genera, *Atrichum*, *Pogonatum*, and *Itatalia*.

### Structure of Early Divergent Moss Capsules and Comparisons With Early Fossil Plants

**FIGURE 3:**
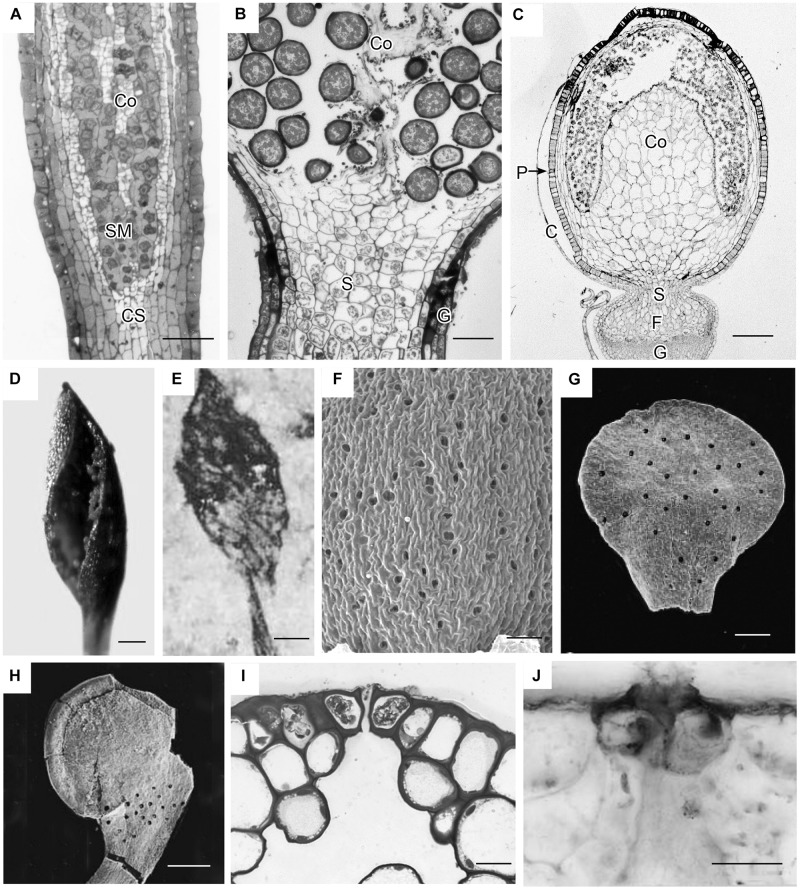
Capsule anatomy, pseudostomata and stomata in extant members of early divergent moss lineages, and sporangia and stomata of the first fossil land plants. **(A)** *Takakia ceratophylla*. Light micrograph (LM) longitudinal section of solid cylindrical capsule with spore mother cells (SM), columella (Co) and conducting strand (CS) in seta. **(B)** *Andreaea rothii*. LM longitudinal section of solid capsule with spores, columella (Co) and short seta (S) surrounded by gametophyte (G) tissue of the pseudopodium. **(C)** *Sphagnum tenellum*. LM longitudinal section of solid capsule, covered by calyptra (C), with pseudostomata (P) in the epidermis, massive columella (Co) covered by the spore sac, and highly reduced seta (S) embedded by foot (F) into gametophyte (G) pseudopodium. **(D)** *Takakia ceratophylla* capsule with single spiraled suture and spores. **(E)** *Tortilicaulis transwalliensis* capsule from the Silurian resembles *Takakia* in **(D)**. **(F)** *Sphagnum tenellum* SEM showing scattered pseudostomata on dried capsule. **(G)** Early Devonian bivalved sporangium with scattered stomata (spots). **(H)** Early Devonian sporangium with band of stomata (spots) at base. **(I)** *Oedipodium* LM cross section of neck with guard cells with ledges over substomatal cavity. **(J)** *Aglaophyton major* from Rhynie Chert. Cross section of mature axis with stoma showing guard cells with ledges over substomatal cavity. Fossil images reproduced with permission from Journal of Experimental Botany (Edwards et al., 1998) and Paleontology (Edwards, 1979). Bars: **(A,E,H)** = 100 μm; **(B,G,J)** = 50 μm; **(C,F)** = 500 μm; **(D)**= 200 μm, **(I)** = 20 μm.

Capsules of extant mosses in early divergent lineages (*Takakia* and *Andreaea*) lack stomata or contain over 100 pseudostomata that do not form pores and are evenly dispersed across the capsule epidermis (Sphagnales). These capsules lack apophyses, have prominent central columellae and have solid tissue throughout without air spaces (Figures 3A–C). Sporophyes of *Andreaea* and *Sphagnum* have short setae, and are embedded in an elongated pseudopodium of the gametophyte (Figures 3B,C). Comparisons with the oldest fossil plants reveal similar capsule morphology and stomatal arrangement/anatomy as in each of these extant early divergent mosses (Figures 3D–J). Sporangia of *Tortilicaulis* from the Silurian are spiraled and similar in shape to *Takakia* (Figures 3D,E). Early Devonian sporangia approximately 400 million years old demonstrate the occurrence of stomata scattered cross sporangia (Figure 3G), resembling the arrangement of pseudostomata in *Sphagnum* (Figure 3F), and restricted to the base similar to extant mosses (Figure 3H). In section, the stomatal complex of the earliest fossils have guard cells with ledges and substomatal cavities much like those of *Oedipodium*, the first moss group to possess stomata (Figures 3I,J).

### Anatomy of Peristomate Moss Capsules

**FIGURE 4:**
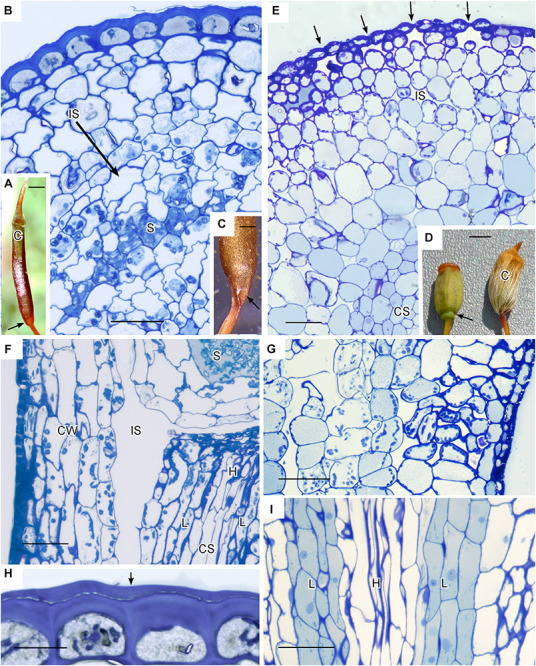
Structure of Polytrichaceae capsule. **(A–C,F,H)** *Atrichum angustatum* that lacks stomata in left hand column. **(D,E,G,I)** *Polytrichastrum ohiensis* with approximately 100 stomata in right hand column. **(A)** Long cylindrical red-brown mature *Atrichum* capsule with inconspicuous calyptra (C) on the top and tapering neck region (arrow) connecting to seta. **(B)** LM cross section at the capsule urn showing solid capsule wall, developing sporogenous region (S) and circumsporangial space (IS) forming between the capsule wall and spore sac. **(C)** Base of recently opened *Atrichum* capsule showing constriction of neck region (arrow) due to drying in circumsporangial cavity and connecting space. **(D)** Two mature *Polytrichastrum* capsules, left without calyptra and right covered by calyptra (C). The capsule is wide and green at the base where the calyptra ends and the narrowly constricted area of the apophysis houses stomata (arrow). **(E)** LM cross section at the constriction with multiple stomata (arrows), subtended by substomatal cavities and associated intercellular spaces (IS), and central conducting strand (CS). **(F)** LM longitudinal section at the junction between spore sac with spores (S) and neck. A large circumsporangial space (IS) extends just inside the solid capsule wall (CW), along the length of the spore sac and downward into the neck. The conducting strand (CS) of hydroids (H) and leptoids (L) ends abruptly at the circumsporangial space and spore sac. **(G)** LM longitudinal section at the constriction showing chloroplast rich cells next to spaces associated with substomatal region on the right and the circumsporangial space to the far left. **(H)** Epidermis with thick walls and cuticle (arrow). **(I)** Prominent conducting strand in the apophysis with leptoids (L) around hydroids (H). Bars: **(A)** = 0.5 mm, **(B,E–G,I)** = 50 μm, **(C)** = 0.2 mm, **(D)** = 1.0 mm, **(H)** = 20 μm.

Members of the Polytrichaceae have well-developed capsule regardless of whether they lack stomata (represented by *Atrichum*) or contain stomata (represented by *Polytrichastrum* with 100+ stomata) (Figure 4). In *Atrichum* the capsule is brown (reddish) when mature and cylindrical, and the short calyptra is situated at the apex (Figure 4A). From the urn down, the neck tapers toward the seta and there is no distinct apophysis. In *Polytrichastrum*, the capsule is swollen throughout with extensive internal spaces (Figures 4D,E). The distinct apophysis is green with a constriction at the base where the stomata are located. The calyptra covers the capsule up to the constriction throughout development. Side-by-side sections illustrate the arrangement of tissues, including air spaces, in these closely related genera. *Atrichum* lacks peripheral spaces including substomatal cavities (Figure 4B) that are abundant in *Polytrichastrum* (Figure 4E). Chloroplasts line cells associated with substomatal cavities (Figure 4G). A large internal air space occurs in *Atrichum* at the base of the capsule and around the entire spore sac (Figure 4F). This circumsporangial space forms in the young capsule just interior to the solid capsule wall in a zone between the amphithecium and endothecium, the two primary embryonic regions (Figure 4B). In both genera, a well-developed conducting strand of hydroids and leptoids extends in the seta to the spore sac where it ends abruptly and presumably fills the internal space with water and nutrients (Figures 4F,I). Unlike *Polytrichastrum* that has stomata to draw water toward the outside, the apophysis of *Atrichum* is covered in a thick cuticle, which retards water loss through the epidermis (Figure 4H). Capsule dehiscence through detachment of the operculum follows drying of liquid in the circumsporangial space and the constriction of the neck at the capsule base (Figure 4C).

### Types and Development of Intercellular Spaces

**FIGURE 5:**
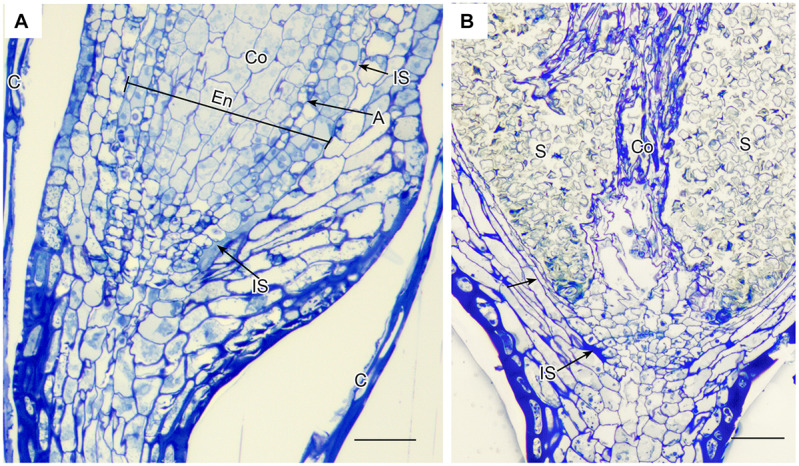
*Leucobryum glaucum*. LM longitudinal sections of astomate capsule. **(A)** Base of immature capsule where seta meets the neck covered by calyptra (C). An inconspicuous fluid-filled intercellular space (IS) extends the entire length of the region between the amphithecium that forms the capsule wall, and the endothecium (En) that consists of a prominent columella (Co) and developing spore sac with one layer of archesporium (A) (sporogeneous tissue). **(B)** Fully expanded capsule. With development of the spore sac that contains 100s of spores (S), the columella (Co) has partially degenerated and the intercellular spaces are closed (arrow) or residual (IS). Bars = 25 μm.

**FIGURE 6:**
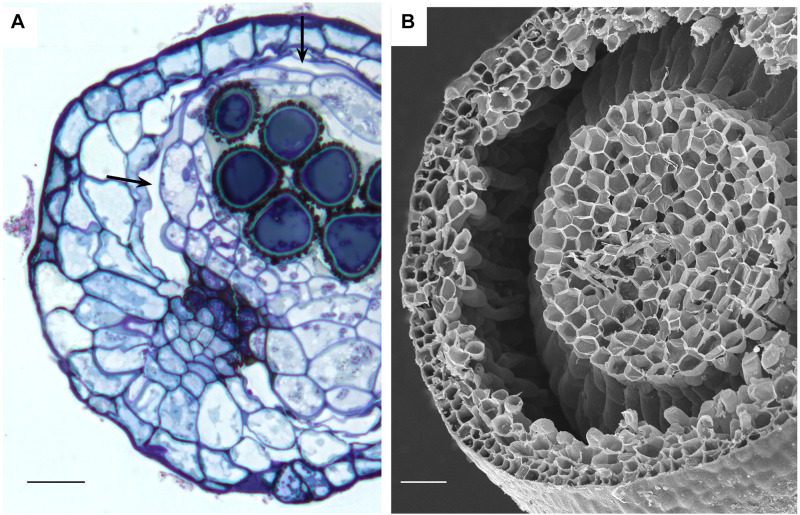
Stomata-containing capsules showing internal circumsporangial space (arrows) that forms between the embryonic endothecium and amphithecium, extends into the neck, and is involved in hydrating and nourishing the spore sac during development. **(A)** LM *Ephemerum*. **(B)** SEM *Plagiomnium*. Image credit: Jeffrey J. Duckett. Bars: **(A)** = 35 μm; **(B)** = 50 μm.

Anatomy of capsules with and without stomata reveals two types of intercellular spaces: (1) the substomatal cavity and connected spaces associated with stomata and (2) the circumsporangial cavity that surrounds the spore sac and may extend into the capsule neck and seta (Figures 4, 5, 6). No stomata-lacking capsules have substomatal cavities and associated spaces but all capsules of peristomate mosses examined in this study possess circumsporangial cavities, regardless of whether they have stomata or not (Figures 4F, 5, 6). As illustrated in *Atrichum* (Figure 4F), *Ephemerum* (Figure 6A) and *Brachythecium* (Figure 6B), circumsporangial cavities surround the developing sporogenous tissue and are intimately associated with conducting tissue (when present), which delivers water and food to the developing spores. In some mosses that lack stomata, like *Leucobryum*, this circumsporangial space is found only during capsule development (Figure 5). The circumsporangial space forms between the embryonic endothecium and amphithecium, prior to the proliferation of sporogenous tissue, and extends the length of the spore sac when the archesporium is a single cell layer (Figures 4A, 5A). When the capsule is fully developed in taxa like *Leucobryum*, no space is discernible due to capsule expansion and spore differentiation (Figure 5B).

**FIGURE 7:**
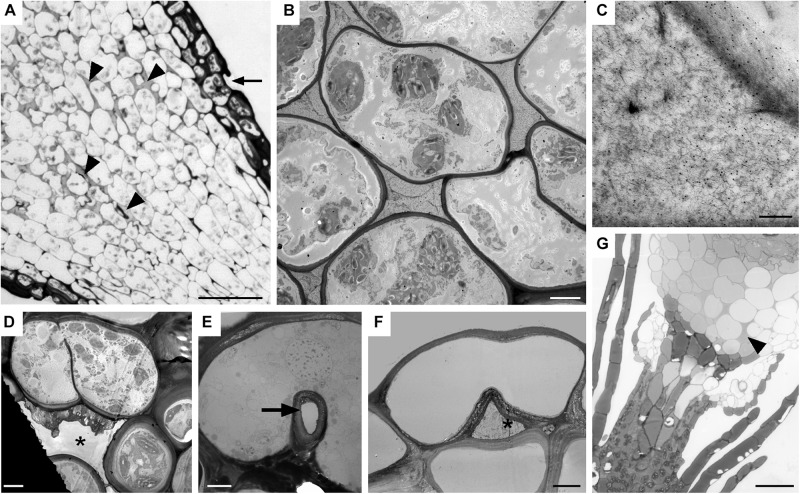
Substomatal cavities and intercellular spaces. **(A–D)** *Dicranum scoparium*. **(A)** LM tangential section of expanding capsule showing stomata (arrow) and associated intercellular spaces are liquid-filled (arrow heads). **(B)** TEM of circumsporangial space filled with dense filamentous material. **(C)** Immunogold labeling TEM shows the liquid in the developing circumsporangial space is positive for the LM19 antibody that recognize homogalacturonan pectin (small black dots). **(D)** Substomatal cavity begins to form before pore opening. **(E,F)** TEM micrographs of *Physcomtrium patens.* **(E)** Small round pore (arrow) of the single-celled stoma. **(F)** Reduced substomatal cavity^∗^. **(G)** LM of liquid-filled intercellular spaces (arrowhead) that are part of the circumsporangial space and not associated with the epidermis of a *P. patens* class 1 KNOX mutant that lacks stomata. Bars: **(A)** = 25 μm; **(B,D–F)** = 4 μm; **(C)** = 200 nm; **(G)** = 20 μm.

Both types of spaces, substomatal cavities and circumsporangial spaces form in the spear stage just before capsule expansion in mosses with stomata. In taxa with stomata, stomata and liquid-filled substomatal cavities form in the expanding neck or apophysis before the sporogenous tissue develops (Figure 7A). Circumsporangial spaces are not associated with stomata and are found in all mosses during development. They begin with the deposition of an electron-dense fibrillar material (Figure 7B) that abundantly localizes with the monoclonal antibody LM19, which recognizes unesterified homogalacturonas pectin (Figure 7C). When the capsule begins to expand and spaces become larger, the fluid inside the space lacks substructure and no longer localizes with this antibody (Merced and Renzaglia, 2016). Substomatal cavities, in contrast, do not form in the absence of stomata and do not label with LM19 early in development (not shown). Their development is coordinated with differentiation of the guard mother cell and before the division of guard cells and pore opening (Figure 7D). The doughnut shaped guard cell of *P. patens* has a small round pore (Figure 7E) and a very reduced substomatal cavity (Figure 7F). *P. patens* sporophytes without stomata have no substomatal cavities but the more internal liquid-filled intercellular spaces are connected to the circumsporangial space and remain throughout development (Figure 7G).

## Discussion

Extant members of early divergent moss lineages entirely lack stomata (Takakiales and Andreaeales) or contain pseudostomata as in Sphagnales. Pseudostomata are pairs of specialized epidermal cells that lack cell wall ledges, do not completely separate to form pores and do not have underlying cavities. They collapse when mature, facilitating drying, capsule dehiscence and spore dispersal, and have been interpreted as either independent from stomata in origin (Duckett et al., 2009) or as modified stomata (Renzaglia et al., 2007; Merced, 2015; Merced and Renzaglia, 2017). Capsule anatomy in these three ancient lineages reflects the absence of pores as intercellular spaces are lacking and the capsule wall and columella are solid throughout. All of these distinctive capsules are erect, lack peristomes, do not contain a distinctive neck or swollen capsule base (apophysis) where stomata are housed, and disperse spores simultaneously with capsule dehiscence through sutures. The early divergent mosses universally lack pore-producing stomata. This includes the Sphagnales that produce high numbers of pseudostomata (100–200 per capsule) that have been interpreted as either independent from stomata in origin (Duckett et al., 2009) or modified stomata (Merced, 2015; Merced and Renzaglia, 2017). Capsule anatomy reflects the absence or pores as intercellular spaces are lacking in *Takakia, Andreaea*, and *Sphagnum* and the capsule wall and columella are solid throughout. All of these distinctive capsules are erect, lack peristomes, do not contain a swollen capsule base (apophysis) or distinctive neck where stomata are housed, and disperse spores simultaneously with capsule dehiscence through sutures. Across mosses, the capsules of *Sphagnum* and *Andreaea* (and Andreaeobryum not studied here) are uniquely positioned on a gametophytic extension or pseudopodium, not a sporophytic seta, and both generations lack conducting tissues. *Takakia* resembles other mosses in that gradual seta elongation elevates the capsule and there is a strand of water conducting cells that ends at the capsule base, albeit the cells in the strand are fundamentally different in development, and structure from those of moss hydroids (Renzaglia et al., 1997, 2000, 2007). These unique architectural features preclude comparisons with more derived peristomate mosses and suggest that true stomata evolved after mosses diversified (Duckett and Pressel, 2018). Consequently, we turned to the fossil record for clues as to when in moss evolution stomata evolved.

Because bryophytes exclusively bear stomata on sporangia, we surveyed the literature on the oldest fossil land plants with reference to sporangia and the occurrence, structure and anatomy of stomata. Fossil plants from the Silurian and early Devonian demonstrate that the range of variability in sporangia seen in extant mosses existed approximately 400 million years ago. These earliest fossil sporangia both bore stomata and lacked stomata, e.g., *Tortilicaulis*, which has a twisted sporangium that is remarkable similar to *Takakia* (Renzaglia et al., 1997, 2017; Edwards et al., 1998). The oldest fossil sporangia were valvate and contained stomata evenly dispersed on the surface similar to pseudostomata of *Sphagnum*, or aggregated at the base in a location that is reminiscent of those on moss necks and apophyses. Details of fossil stomata reveal guard cells and internal anatomy similar to that in *Oedipodium*, the first moss lineage with stomata. Based on the existence of stomata on sporangia in the first plant macrofossils and the similarities with architectural features of early mosses, it is quite possible/likely that stomata existed on moss capsules prior to the diversification of peristomate mosses, which occurred over 100 million years after mosses originated (Newton et al., 2009). Indeed, the estimated median stem age of *Takakia* and *Sphagnum* based on the oldest fossil land plants is 465 Ma, while those for *Tetraphis* and *Oedipodium* are 309 and 298 Ma, respectively (Laenen et al., 2014). This line of evidence identifies stomata on sporangia that resemble moss capsules when stomata first appeared in the fossil record. Early plant fossils and the high incidence of stomata loss in extant mosses are consistent with the hypothesis that stomata evolved once in bryophytes and were lost repeatedly during diversification, including in early divergent lineages and along the entire moss phylogeny.

Losses of stomata in peristomate mosses are numerous and widespread throughout acrocarps and pleurocarps (Figure 1). Minimally we identify 40 families and 74 genera in which stomata are absent. Of these, 63 are estimated to be independent losses based on phylogenetic relationships. This is a low estimate given the scant record of descriptions and counts of stomata in mosses. Stomata are first seen in the Oedipodiaceae, Tetraphidaceae and Polytrichaceae. The first family includes the single genus *Oedipodium*, which has the most elongated neck found in any moss and contains approximately 60 stomata (Shaw and Renzaglia, 2004). Both genera in the Tetraphidaceae have erect cylindrical capsules with simple anatomy and minimal neck. *Tetrodontium* contains five stomata while *Tetraphis* has none and has an anatomy at the short neck that is devoid of air spaces.

With 3–30 stomata in 74% of moss families (40 of the 54 families based on published counts), stomatal numbers per capsule are relatively low in most mosses. Only 9% of families with counts have more than 100 stomata per capsule. Four families include no members with stomata. Even in the groups with high numbers of stomata there are species with single digit to zero stomata. In general, higher numbers of stomata are found in sporophytes with larger capsules, but capsules devoid of stomata are variable in size (Paton and Pearce, 1957). In the Polytrichaceae, for example, stomata-free capsules of *Atrichum* and *Pogonatum* are similar in length to those of *Polytrichum*, which has up to 250 stomata per capsule (Smith Merrill, 2007). Ecological factors do not explain the absence of stomata either as these taxa often occur side by side along forest floors. In some cases, losses of stomata appear to be associated with capsule reduction. For example, in the Pottiaceae, a transformational series of capsule and seta reduction is associated with high incidences of stomatal losses that have been reported in eight genera (Zander and Eckel, 1993). In other instances, stomatal numbers are relatively low but no known instances of loss have been documented. In the Orthotrichaceae, for example, capsules that are immersed in protective leaves still possess stomata (Merced and Renzaglia, 2017) and cleistocarpic capsules of *Ephemerum* and *P. patens* also have stomata (Merced and Renzaglia, 2013). A clear trend is the absence of stomata in aquatic bryophytes, e.g., *Fissidens* subg. *Octodiceras* and *Fontinalis* (Supplementary Data) or semi aquatic taxa when submerged.

Anatomy and development are foundational for understanding plant structure/function relationships and evolution. Our examinations of the internal organization of tissues and their development in capsules confirm that the mosses in early divergent lineages, *Takakia, Andreaea* and Sphagnaceae, lack any type of intercellular space in the sterile tissue of the capsule, and that peristomate mosses possess intercellular spaces some time in development even if stomata are absent (Duckett and Pressel, 2018). Although these spaces in mosses begin development with the secretion of a fluid-filled matrix, we demonstrate the existence of two distinct types of intercellular spaces in moss capsules. The first is the substomatal cavity associated only with stomata. The second is a circumsporangial space that extends between the spore sac and capsule wall and is involved in capsule expansion during sporogenesis. In many capsules with stomata such as *Funaria*, circumsporangial spaces extend into the apophysis and eventually connect with substomatal cavities, forming an elaborate system of internal spaces (Merced and Renzaglia, 2016).

We identify different origins for the two types of intercellular spaces in moss capsules. Substomatal cavities begin to develop at the spear stage in concert with guard cell differentiation before sporogenesis. The formation of substomatal cavities involves deposition of a fluid in the cavity that does not localize for pectins, suggesting it is not mucilaginous in nature (Merced and Renzaglia, 2014, 2016). These cavities are necessary for guard cells to separate, develop their unique walls, and for the pore to form. The extent of the system of substomatal cavites and circumsporangial space is related to the size of the capsule or apophysis where stomata are present. This is exemplified in the large capsules of *Oedipodium, Funaria*, and *Polytrichum* with extensive interconnected systems of substomatal cavities and underlying intercellular spaces versus the reduced capsules of *Ephemerum* and *P. patens* that have small substomatal cavities and a reduced circumsporangial space (Merced and Renzaglia, 2013, 2014, 2016). No mosses without stomata, including stomata free mutants of *P. patens*, form cavities directly beneath the epidermis that compare with substomatal cavities. Similarly, the absence of substomatal cavities in Sphagnales coincides with the absence of pores in pseudostomata.

In tracheophytes, stomata and intercellular spaces are coordinated throughout development to maximize gas exchange and minimize water lost. The molecular mechanisms controlling air spaces and stomata placement are now being elucidated, and it is hypothesized that feedback signaling between stomata and air spaces influences mesophyll arrangement (Baillie and Fleming, 2020). In mutant wheat plants with arrested stomata, when guard cells fail to divide and do not form a pore, no substomatal cavity is formed (Lundgren et al., 2019). This is similar to what we observed in mosses without stomata, i.e., that substomatal cavities fail to form. The loss of pore formation in *Sphagnum* and lack of intercellular spaces is consistent with an interpretation that pseudostomata are modified stomata (Merced, 2015).

Unlike substomatal cavities, circumsporangial spaces form in all capsules of peristomate mosses regardless of whether they have stomata or not. This internal space develops with the deposition of fluid that results in an expanding schism between capsule wall and spore sac. As illustrated in the immature *Leucobryum* and mature *Atrichum, Ephemerum* and *Plagiomnium* capsules, the circumsporangial space extends around the entire spore sac, providing a protective and nutritive matrix during spore differentiation. Unlike substomatal cavities, the fluid in this internal space contains pectins as labeled by the LM19 antibody, suggestive of mucilage, and evidence that the two types of spaces are developmentally and genetically independent. The antibody LM19 recognize epitopes of unesterified homogalacturonan, pectin, a polymer found in cell walls of all land plants that is an important component of guard cell walls and mucilage of bryophytes and angiosperms (Merced and Renzaglia, 2014, 2019; Renzaglia et al., 2017).

The separation zone that forms the circumsporangial space is determined in the formative stage of embryogenesis at the time of delineation of the endothecium, which develops into the spore sac plus columella, and amphithecium that forms the capsule wall (Smith, 1955). In comparison, intercellular spaces in hornwort sporophytes are associated with stomata only and are therefore lacking in the two hornwort clades that have lost stomata (Renzaglia et al., 2017). That no circumsporangial space occurs in hornworts is easily explained by the continued and gradual maturation of the cylindrical sporophyte from a basal meristem upward. Unlike in mosses, there is no massive capsule expansion in width in hornworts. Moreover, formative divisions in hornworts do not mimic those in mosses as the amphithecium gives rise to the sporogenous tissue and endothecium, while the endothecium produces only the columella (Renzaglia, 1978). Developmentally there are few similarities between moss and hornwort sporophytes, thus stomata loss is associated with different anatomical modifications in the two bryophyte clades.

The astomate capsule of *Atrichum* provides abundant clues to the potential role of the internal spaces in moss capsules. In this plant, large spaces remain around and below the spore sac throughout development. These are fluid-filled from their origin and dry following capsule expansion and spore maturation. The circumsporangial spaces are strategically positioned around and above the sporogenous tissue at the region where the central strand of conducting tissue abruptly ends in the neck. The neck in turn consists of tightly packed cells with an epidermis covered by a thick cuticle. Based on this architecture, it is reasonable to deduce that water and dissolved photosynthate that is drawn up to the top of the neck fills the space around the spore sac. In this arrangement, sporogenous tissue is hydrated and provided with a constant source of nutrients. Unlike the neck or apophysis of stomata-containing mosses, there is no potential for a transpirational pull of water up and out of the capsule. Rather, water and solutes are sequestered around the developing spores, and resources are utilized and replenished as needed. This path from source to sink is unidirectional and draws nutrients and water from the gametophyte through the placenta and into the capsule throughout differentiation. The greater loss of water in astomate *Atrichum* capsules than in stomata bearing taxa as reported by Duckett and Pressel (2018) can be explained by the directed and constant use of water and nutrients in this closed systems. In the final stages of capsule differentiation, the fluid dries in the circumsporangial space, compressing the capsule urn and neck, and resulting in the detachment of the operculum and progressive spore release throughout the season.

A dearth in developmental and structural studies of moss capsules has limited comparisons across the group, making the role of specific anatomical structures in capsule function difficult to interpret. For example, there are many genera for which stomata occurrence and counts are not recorded. The existence and arrangement of key tissues such as conducting tissue are not adequately documented. Consequently, it is not verified but only speculated that hydroids occur in most moss setae (Hébant, 1977). There are mosses such as *Orthotrichum* that possess stomata but do not have conducting tissue in the sporophyte. *Grimmia*, in contrast, has been reported to have conducting tissue in the gametophyte but none in the sporophyte, while *Buxbaumia* has hydroids but no leptoids solely in the sporophyte. How these anatomical differences impact nutrient movement and capsule function are in need of further studied.

Coupled with our morphological and anatomical observations, recent studies on physiology and genetics are providing a comprehensive picture of function and evolution of stomata in bryophytes (Chater et al., 2017). Moss and hornwort stomata do not respond to environmental and endogenous cues including light intensity, water status, abscisic acid, plasmolysis, and physical damage as do angiosperm stomata (Pressel et al., 2018). In bryophytes there are no mechanisms for stomatal pores to open and close and ion changes are the same in all epidermal cells (Sussmilch et al., 2019). The preponderance of recent evidence suggests that stomata play a strategic role in capsule maturation, drying, and dehiscence without any active regulation of water loss.

The function of moss capsules in nourishing, hydrating, protecting, and dispersing spores occurs regardless of whether stomata are present. Stomata have been eliminated in over 60 moss genera/lineages in capsules that are highly modified in anatomy compared with their stomata-bearing relatives. The repeated and numerous evolutionary events that reduced and eliminated stomata on moss capsules point to the fact that unlike in tracheophytes where stomata loss is rare and restricted in occurrence (Keeley et al., 1984; Woodward, 1998), stomata are not necessary for mosses. The loss of stomata has no major consequences for the physiology of the sporophyte but results in delayed maturation and dispersion of spores in stomata-less mutants of *P. patens* (Chater et al., 2016, 2017). Capsule architecture in mosses without stomata ranges from solid in taxa in early divergent lineages to containing an internal circumsporangial space that is directly connected to the conducing tissue and is involved in capsule expansion and the nourishment, hydration and development of spores. This anatomy reveals there are different architectural arrangements of tissues within moss capsules that are equally effective in accomplishing the essential processes of sporogenesis and spore dispersal. Stomata are not foundational to these processes.

## Data Availability Statement

All datasets generated for this study are included in the article/Supplementary Material.

## Author Contributions

KR designed the study, conducted anatomical studies, prepared the figures, analyzed the data, and wrote the manuscript. AM conducted ultrastructural studies/immunogold labeling, generated the phylogenetic tree and assisted in preparing the figures and writing the manuscript. WB assisted with generating the phylogenetic tree, conducted literature searches, compiled data tables, and assisted in anatomical studies. All authors read and approved the manuscript.

## Conflict of Interest

The authors declare that the research was conducted in the absence of any commercial or financial relationships that could be construed as a potential conflict of interest.

## References

[B1] Abella L. Alcalde M. Estébanez B. Cortella A. Alfayate C. Ron E. (1999). Observations on the stomatal complex in ten species of mosses (Pottiaceae, Bryopsida). *J. Hattori Bot. Lab.* 86, 179–185 10.18968/jhbl.86.0_179

[B2] Allen B. (2015). “Fontinalaceae,” in *Flora of North America North of Mexico*, Vol. 28 ed. Flora of North America Editorial Committee (New York, NY: Oxford University Press), 489–494.

[B3] Andreas B. K. (2013). A revision of *Blindia* (*Seligeriaceae*) from Southern South America. *Bryol.* 116 263–280. 10.1639/0007-2745-116.3.263

[B4] Arzeni C. B. (1954). The Pterobryaceae of the Southern United States, Mexico, Central America, and the West Indies. *Am. Midl. Nat.* 52 1–67.

[B5] Baillie A. L. Fleming A. J. (2020). The developmental relationship between stomata and mesophyll airspace. *New Phytol.* 225 1120–1126. 10.1111/nph.16341 31774175

[B6] Bartlett J. K. Vitt D. H. (1986). A survey of species in the genus *Blindia* (Bryopsida, Seligeriaceae). *N. Z. J. Bot.* 24 203–246. 10.1080/0028825X.1986.10412674

[B7] Bryan V. S. (2007). “Ephemeraceae,” in *Flora of North America North of Mexico*, Vol. 2 ed. Flora of North America Editorial Committee (New York, NY: Oxford University Press), 646–649.

[B8] Buck W. R. (1981). A re-interpretation of the Fabroniaceae, III: anacamptodon and Fabronidium revisited, Mamillariella, Helicodontiadelphus and Bryobartlettia gen. nov. *Brittonia* 33 473–481.

[B9] Chater C. C. Caine R. S. Fleming A. J. Gray J. E. (2017). Origins and evolution of stomatal development. *Plant Phys.* 174 624–638. 10.1104/pp.17.00183 PMC5462063 28356502

[B10] Chater C. C. Caine R. S. Tomek M. Wallace S. Kamisugi Y. Cuming A. C. (2016). Origin and function of stomata in the moss *Physcomitrella patens*. *Nat. Plants* 2 1–7. 10.1038/nplants.2016.179 PMC5131878 27892923

[B11] Cheney L. S. (1897). North American species of Amblystegium. *Bot. Gazette* 24 236–291. 10.1086/327591

[B12] Crum H. A. Anderson L. E. (1981). *Mosses of Eastern North America.* New York, NY: Columbia University Press.

[B13] Dow G. J. Berry J. A. Bergmann D. C. (2017). Disruption of stomatal lineage signaling or transcriptional regulators has differential effects on mesophyll development, but maintains coordination of gas exchange. *New Phytol.* 216 69–75. 10.1111/nph.14746 28833173 PMC5601202

[B14] Duckett J. G. Pressel S. (2018). The evolution of the stomatal apparatus: intercellular spaces and sporophyte water relations in bryophytes—two ignored dimensions. *Philos. Trans. R. Soc. Lond. B Biol. Sci.* 373:20160498. 10.1098/rstb.2016.0498 PMC5745334 29254963

[B15] Duckett J. G. Pressel S. P’ng K. M. Renzaglia K. S. (2009). Exploding a myth: the capsule dehiscence mechanism and the function of pseudostomata in *Sphagnum*. *New Phytol.* 183 1053–1063. 10.1111/j.1469-8137.2009.02905.x 19552695

[B16] During H. J. (1977). *A taxonomical Revision of the Garovaglioideae (Pterobryaceae, Musci).* thesis, Bryophytorum Bibliotheca12, J. Cramer Verlag, Vaduz.

[B17] Edwards D. (1979). A late Silurian flora from the lower old red sandstone of south-west dyfed. *Palaeontology* 22 23–52.

[B18] Edwards D. Kerp H. Hass H. (1998). Stomata in early land plants: an anatomical and ecophysiological approach. *J. Exp. Bot.* 49 255–278.

[B19] Egunyomi A. (1982). On the stomata of some tropical African mosses. *Lindbergia* 8 121–124.

[B20] Fife A. J. (2012). New taxa of *Sematophyllum* and Wijkia (Musci:Sematophyllaceae), with a key to New Zealand Sematophyllaceae. *N. Z. J. Bot.* 50 435–447. 10.1080/0028825X.2012.728993

[B21] Fife A. J. Dalton P. J. (2005). A reconsideration of *Pleurophascum* (Musci: *Pleurophascaceae*) and specific status for a New Zealand endemic, Pleurophascum ovalifolium stat. et nom. nov. *N. Z. J. Bot.* 43 871–884. 10.1080/0028825X.2005.9512997

[B22] Goffinet B. (2012). *Australian Mosses Online. 53. Splachnaceae.* Avaliable online at: http://www.anbg.gov.au/abrs/Mosses_online/Splachnaceae.pdf

[B23] Goffinet B. Buck W. R. Shaw A. J. (2009). “Morphology, anatomy and classification of the Bryophyta,” in *Bryophyte Biology*, eds Goffinet B. Shaw A. J., (Cambridge, MA: Cambridge University Press), 55–138.

[B24] Gradstein S. R. Churchill S. P. Salazar-Allen N. (2001). *Guide to the Bryophytes of Tropical America.* The Bronx, NY: Memoirs New York Botanical Garden.

[B25] Hastings R. I. Grevens H. C. (2007). “Grimmia,” in *Flora of North America North of Mexico*, Vol. 27 ed. Flora of North America Editorial Committee (New York, NY: Oxford University Press), 225–257.

[B26] Hattaway R. A. (1984). *A Monograph of the Ptychomniaceae (Bryopsida).* Ph.D. dissertation, Pennsylvania State University, University Park, PA.

[B27] Hébant C. (1977). The conducting tissue of bryophytes. *Bryophyt. Bibl.* 10:157.

[B28] Hedenäs L. (2005). Bryophyte flora of Uganda. 4. Rhytidiaceae, Hylocomiaceae and Hypnaceae (Part 1). *J. Bryol.* 27 55–66. 10.1179/174328205x40734

[B29] Ireland R. (2015). “Plagiotheciaceae,” in *Flora of North America North of Mexico*, Vol. 28 ed. Flora of North America Editorial Committee (New York, NY: Oxford University Press), 483–488.

[B30] Jennings O. E. (1913). *A Manual of the Mosses of Western Pennsylvania.* Mosses, PA: Press of the City Mission Pub. Co., 10.5962/bhl.title.54494

[B31] Keeley J. E. Osmond C. B. Raven J. A. (1984). Stylites, a vascular land plant without stomata absorbs CO2 via its roots. *Nature* 310 694–695.

[B32] Klazenga N. (2012a). *Australian Mosses Online. 28. Orthorrhynchiaceae.* Avaliable online at: http://www.anbg.gov.au/abrs/Mosses_online/Orthorrhynchiaceae.pdf

[B33] Klazenga N. (2012b). *Australian Mosses Online. 29. Pterigynandraceae.* Avaliable online at: http://www.anbg.gov.au/abrs/Mosses_online/Pterigynandraceae.pdf

[B34] Laenen B. Shaw B. Schneider H. Goffinet B. Paradis E. Désamoré A. (2014). Extant diversity of bryophytes emerged from successive post-Mesozoic diversification bursts. *Nat. Commun.* 5:5134. 10.1038/ncomms6134 25346115

[B35] Liu Y. Johnson M. G. Cox C. J. Medina R. Devos N. Vanderpoorten A. (2019). Resolution of the ordinal phylogeny of mosses using targeted exons from organellar and nuclear genomes. *Nat. Commun.* 10:1485 10.1038/s41467-019-09454-w PMC6445109 30940807

[B36] Lundgren M. R. Mathers A. Baillie A. L. Dunn J. Wilson M. J. Hunt L. (2019). Mesophyll porosity is modulated by the presence of functional stomata. *Nat. Commun.* 10:2825. 10.1038/s41467-019-10826-5 PMC6597550 31249299

[B37] McIntosh T. T. (2007). “Schistidium,” in *Flora of North America North of Mexico*, Vol. 27 ed. Flora of North America Editorial Committee (New York, NY: Oxford University Press), 207–224.

[B38] Merced A. (2015). Novel insights on the structure and composition of pseudostomata of *Sphagnum*. *Am. J. Bot.* 102 329–335. 25784466 10.3732/ajb.1400564

[B39] Merced A. Renzaglia K. (2014). Developmental changes in guard cell wall structure and pectin composition in the moss Funaria: implications for function and evolution of stomata. *Ann. Bot.* 114 1001–1010. 10.1093/aob/mcu165 25129633 PMC4171074

[B40] Merced A. Renzaglia K. S. (2013). Moss stomata in highly elaborated *Oedipodium* (Oedipodiaceae) and highly reduced Ephemerum (Pottiaceae) sporophytes are remarkably similar. *Am. J. Bot.* 100 2318–2327. 10.3732/ajb.1300214 24302694

[B41] Merced A. Renzaglia K. S. (2016). Patterning of stomata in the moss *Funaria*: a simple way to space guard cells. *Ann. Bot.* 117 985–994. 10.1093/aob/mcw029 27107413 PMC4866314

[B42] Merced A. Renzaglia K. S. (2017). Structure, function and evolution of stomata from a bryological perspective. *Bryophys. Divers. Evol.* 39 7–20.

[B43] Merced A. Renzaglia K. S. (2019). Contrasting pectin polymers in guard cell walls of Arabidopsis and the hornwort Phaeoceros reflect physiological differences. *Ann. Bot.* 123 579–585. 30202908 10.1093/aob/mcy168 PMC6417473

[B44] Milne J. Klazenga N. (2012). *Australian Mosses Online. 24. Entodontaceae.* Avaliable online at: http://www.anbg.gov.au/abrs/Mosses_Online/Entodontaceae.pdf

[B45] Newton A. E. Wikström N. Shaw A. J. Hedges S. B. Kumar S. (2009). *Mosses (Bryophyta). The Time Tree of Life.* New York: Oxford University Press, 138–145.

[B46] Paton J. A. Pearce J. V. (1957). The occurrence, structure and functions of the stomata in British bryophytes. *Trans. Br. Bryol. Soc.* 3 228–259. 10.1179/006813857804829560

[B47] Pressel S. Renzaglia K. S. Clymo R. S. Duckett J. G. (2018). Hornwort stomata do not respond actively to exogenous and environmental cues. *Ann. Bot.* 122 45–57. 10.1093/aob/mcy045 29897395 PMC6025193

[B48] Puttick M. N. Morris J. L. Williams T. A. Cox C. J. Edwards D. Kenrick P. (2018). The interrelationships of land plants and the nature of the ancestral embryophyte. *Curr. Biol.* 28 733–745. 10.1016/j.cub.2018.01.063 29456145

[B49] Pursell R. A. (1987). A taxonomic revision of *Fissidens* subgenus *Octodiceras* (Fissidentaceae). *Mem N. Y. Bot. Gard.* 45 639–660.

[B50] Pursell R. A. (2007). “Fissidentaceae,” in *Flora of North America North of Mexico*, Vol. 27 ed. Flora of North America Editorial Committee (New York, NY: Oxford University Press), 331–357.

[B51] Pursell R. A. Bruggeman-Nannenga M. A. Allen B. H. (1988). A taxonomic revision of *Fissidens* subgenus *Sarawakia* (Bryopsidae: Fissidentacaea). *Bryologist* 91, 202–213.

[B52] Renzaglia K. S. (1978). A comparative morphology and developmental anatomy of the Anthocerotophyta. *J. Hattori Bot. Lab.* 44 31–90.

[B53] Renzaglia K. S. Duff R. J. Nickrent D. L. Garbary D. J. (2000). Vegetative and reproductive innovations of early land plants: implications for a unified phylogeny. *Philos. Trans. R. Soc. Lond. B Biol. Sci.* 355 769–793. 10905609 10.1098/rstb.2000.0615 PMC1692784

[B54] Renzaglia K. S. McFarland K. D. Smith D. K. (1997). Anatomy and ultrastructure of the sporophyte of *Takakia ceratophylla* (Bryophyta). *Am. J. of Bot.* 84 1337–1350. 21708543

[B55] Renzaglia K. S. Schuette S. Duff R. J. Ligrone R. Shaw A. J. Mishler B. D. (2007). Bryophyte phylogeny: advancing the molecular and morphological frontiers. *Bryologist* 110 179–213.

[B56] Renzaglia K. S. Villareal Aguilar J. C. Garbary D. J. (2018). Morphology supports the setaphyte hypothesis: mosses plus liverworts form a natural group. *Bryophys. Divers. Evol.* 40 11–17.

[B57] Renzaglia K. S. Villarreal J. C. Piatkowski B. T. Lucas J. R. Merced A. (2017). Hornwort stomata: architecture and fate shared with 400-million-year-old fossil plants without leaves. *Plant Physiol.* 174 788–797. 10.1104/pp.17.00156 28584065 PMC5462037

[B58] Schofield W. B. (2007). “Diphysciaceae,” in *Flora of North America North of Mexico*, Vol. 27 ed. Flora of North America Editorial Committee (New York, NY: Oxford University Press), 162–164.

[B59] Shaw A. J. Renzaglia K. S. (2004). Phylogeny and diversification of bryophytes. *Am. J. Bot.* 91, 1557–1581. 10.3732/ajb.91.10.1557 21652309

[B60] Smith G. M. (1955). *Cryptogamic Botany: Vol. II Bryophytes and Pteridophytes.* New York, NY: McGraw-Hill Book Company.

[B61] Smith A. J. (2004). *The Moss Flora of Britain and Ireland.* Cambridge: Cambridge University Press.

[B62] Smith Merrill G. L. (2007). “Polytrichaceae,” in *Flora of North America North of Mexico*, ed. Flora of North America Editorial Committee, (New York, NY: Oxford University Press), 121–160.

[B63] Spence J. R. (2015). “Leskeaceae,” in *Flora of North America North of Mexico*, Vol. 28 ed. Flora of North America Editorial Committee (New York, NY: Oxford University Press), 340–361.

[B64] Stark L. (2015). “Leptodontaceae,” in *Flora of North America North of Mexico*, Vol. 28 ed. Flora of North America Editorial Committee (New York, NY: Oxford University Press), 623–627.

[B65] Sussmilch F. C. Roelfsema M. R. G. Hedrich R. (2019). On the origins of osmotically driven stomatal movements. *New Phyt.* 222 84–90. 10.1111/nph.15593 30444541

[B66] Tangney R. S. (1997). A generic revision of the Lembophyllaceae. *J. Hattori Bot. Lab* 81 123–153.

[B67] Tong C. He S. (2002). “Ditrichaceae,” in *Moss Flora of China*, Vol. 1 eds Peng-cheng, Crosby W. He S. (St. Louis, MO: Missouri Botanical Garden Press), 58–80.

[B68] Vitt D. H. (2007). “Drummondia,” in *Flora of North America North of Mexico*, Vol. 27 ed. Flora of North America Editorial Committee (New York, NY: Oxford University Press), 40–41.

[B69] Vitt D. H. Buck W. R. (1984). The familial placement of *Bryowijkia* (Musci: Trachypodaceae). *Brittonia* 36 300–306. 10.2307/2806531

[B70] Woodward F. I. (1998). Do plants really need stomata? *J. Exp. Bot.* 49 471–480.

[B71] Yu N.-N. Jia Y. (2012). The taxonomic status of two species of *Calyptothecium* Mitt. (Pterobryaceae, Bryopsida). *J. Bryol.* 34 63–65. 10.1179/1743282011Y.0000000044

[B72] Zander R. H. Eckel P. M. (1993). *Genera of the Pottiaceae: Mosses of Harsh Environments.* Buffalo, NY: Buffalo Society of Natural Sciences.

