# Supplementary material for: With Over 60 Independent Losses, Stomata Are Expendable in Mosses
Source: Front Plant Sci. 2020 May 28;11:567. doi: 10.3389/fpls.2020.00567 (PMC7270291; doi:10.3389/fpls.2020.00567)
Supplement: Supplementary file 1 [file Data_Sheet_1.doc]

Supplementary Material

# Supplementary Data

74 moss genera (bold) without stomata, organized by order and family. Specie­­s lacking stomata are listed for those genera that also contain stomata-containing species. Sphagnales is not included because they possess pseudostomata. The classification is according to Goffinet and Buck 2019.

ORDER TAKAKIALES

Takakiaceae: ***Takakia***

ORDER ANDREAEALES

Andreaeaceae: ***Andreaea, Acroschisma***

ORDER ANDREAEOBRYALES

Andreaeobryaceae: ***Andreaeobryum***

ORDER POLYTICHALES

Polytichaceae: ***Atrichum, Pogonatum, Itatalia***

ORDER TETRAPHIDALES

Tetraphidaceae: ***Tetraphis***

ORDER DIPHYSCIALES

Diphysciaceae: ***Diphyscium*** *mucronifolium*

ORDER DISCELIALES

Disceliaceae: ***Discelium***

SUBCLASS DICRANIDAE

Catoscopiaceae: ***Catoscopium***

ORDER SCOULERIALES

Scouleriaceae: ***Scouleria***

Drummundiaceae: ***Drummondia***

ORDER: GRIMMIALES

Grimmiaceae: ***Grimmia*** *montana, G. alpestris, G. atrata. G.hamulosa, G. mariniana, G. reflexidens, G. nevadensis, G. serrana,* ***Schistidium*** *occidentale S. atrofuscum, S. crassipilum S. crassipilum, S. cinclidodonteum, S. atrichum, S. agassizii*

Seligeriaceae: ***Blindia*** *seppeltii, B. magellanica, B. martini, B. lewinskyae, B. immerse,*

*B. contecta, B. buckii, B. rigida, B. serrate, B. torrentium B. inundata,* ***Trochobryum****,*

***Seligeria*** *carniolica*

ORDER ARCHIDIALES

Archidiaceae: ***Archidium***

ORDER DICRANALES

Fissidentaceae: ***Fissidens*** *subglaucissimus, Fissidens subg. Octodiceras, F.grandifron*

Ditrichaceae: ***Garckea*** *flexuosa,* ***Bryomanginia****,* ***Crumuscus****,* ***Cygniella***

Bruchiaceae: ***Pringleella*** *sinesis*

Erpodiaceae: ***Erpodium*** *glaucum,* ***Aulacopilum***

Schistostegaceae: ***Schistostega*** *pennata*

Dicranaceae: ***Dicranella*** *heteromalla, D. hawaica,* ***Holomitriopsis****,* ***Wardia*** *hygrometrica*

Micromitriaceae: ***Micromitrium*** *austinii, M. synoicum, M. tenerum*

Leucobryaceae: ***Dicranodontium*** *denudatum,* ***Campylopu****s,* ***Leucobryum****,* ***Brothera****,* ***Ochrobryum****,* ***Pilopogon****,* ***Atractylocarpus****,* ***Microcampylopus*** *laevigatus*

Calymperaceae*:* ***Calymperopsis*** *martinicensis,* ***Calymperes*** *subdecolorans*

ORDER POTTIALES

Pottiaceae: ***Cinclidotus*** *fontinaloides, C. nigricans, C. mucronatus,* ***Nanomitrium****,* ***Aschisma****,* ***Leptodontiella****,* ***Leptodontium****,* ***Trachyodontium****,* ***Streptotrichum****,* ***Trichostomum****,* ***Splachnobryum*** *rostratum*

Pleurophascaceae: ***Pleurophascum*** *occidentale*

ORDER SPLACHNALES

Splachnaceae: ***Tayloria*** *tasmanica*

ORDER HEDWIGIALES

Hedwigiaceae: ***Bryowijkia*** *ambigua, B.* ambigua

Order: HYPNODENDRALES

Pterobryellaceae: ***Pireella***

ORDER PTYCHOMNIALES

Orthorrhynchiaceae: ***Orthorrhynchium***

Rhabdodontiaceae: ***Glyphothecium*** *gracile*

Ptychomniceae: ***Endotrichellopsis*** *laevifolia*

ORDER HOOKERIALES

Pilotrichaceae: ***Cyclodictyon***

ORDER HYPNALES

Fontinalaceae**: *Fontinalis***

Leskeaceae: ***Mamillariella*** *geniculate*

Stereophyllaceae: ***Stereophyllum*** *andogense*

Plagiotheciaceae: ***Platydictya*** *minutissima*

Entodontaceae: ***Mesonodon***

Sematophyllaceae: ***Sematophyllum*** *kirkii*

Cryphaeaceae: ***Cyphaea*** *lamyana*, ***Sphaerotheciella***

Leucodontaceae: ***Leucodon***

Pterobryaceae: ***Calyptothecium***

Neckeraceae: ***Neckeropsis***

Leptodontaceae: ***Forsstroemia***

Lembophyllaceae: ***Camptochaete***

Anomodontaceae: ***Anomodon*** *minor, A. viticulosus,* ***Schwetschkeopsis***

# Supplementary Data

**List of references used to compile stomata number and absence of stomata.**

Andreas, B.K. (2013). A Revision of *Blindia* (Seligeriaceae) from Southern South America. The Bryologist 116, 263–280. doi:10.1639/0007-2745-116.3.263

Bartlett, J.K., Vitt D. H. (1986). A survey of species in the genus *Blindia* (Bryopsida, Seligeriaceae), New Zealand Journal of Botany, 24: 203-246. doi:10.1080/0028825X.1986.10412674

Breitwieser I., Brownsey P.J.; Heenan P.B., Nelson W.A., Wilton A.D. eds. (2010) Flora of New Zealand Online. Accessed at www.nzflora.info January, 2020.

Buck, W.R. (1997). Schofieldiella (Hylocomiaceae), a new genus for an old species. The Journal of the Hattori Botanical Laboratory, 82, pp.39-46.

During, H.J. (1977). A taxonomical revision of the Garovaglioideae (Pterobryaceae, Musci). thesis, Groningen / Bryophytorum Bibliotheca 12, Vaduz.

Egunyomi, A. (1982). On the Stomata of Some Tropical African Mosses. Lindbergia 8, 121–124.

Enroth, J., Magill, R.E. (1994). *Neckeropsis pocsii* (Neckeraceae, Musci), a New Species from Comoro Islands. The Bryologist 97, 171. doi:10.2307/3243755

Fife, A.J. (2012). New taxa of *Sematophyllum* and *Wijkia* (Musci:Sematophyllaceae), with a key to New Zealand Sematophyllaceae, New Zealand Journal of Botany, 50:4, 435-447. doi: 10.1080/0028825X.2012.728993

Fife A. J., Dalton P. J. (2005) A reconsideration of *Pleurophascum* (Musci: Pleurophascaceae) and specific status for a New Zealand endemic, *Pleurophascum ovalifolium* stat. et nom. nov., New Zealand Journal of Botany, 43: 871-884. doi:10.1080/0028825X.2005.9512997

Flora of North America Editorial Committee ed. (1993). *Flora of North America North of Mexico: Bryophyta* (Vol. 28). Oxford University Press.

Goffinet B. (2012) Australian Mosses Online. 53. Splachnaceae. <http://www.anbg.gov.au/abrs/Mosses_online/Splachnaceae.pdf>

Goffinet, B. and Buck, W.R. Classification of the Bryophyta. On-line version available at http://bryology.uconn.edu/classification/.

Goffinet, B., Shaw, A.J. (2009). Bryophyte biology. Cambridge University Press, New York.

Gradstein, S.R., Churchill, S.P. and Salazar-Allen, N. (2001). Guide to the bryophytes of tropical America. Memoirs New York Botanical Garden.

Gradstein, S.R., Crum, H.A., Anderson, L.E. (1982). Mosses of Eastern North America. Taxon 31, 136. doi:10.2307/1220606

Hattaway, R. A. (1984). A Monograph of the Ptychomniaceae (Bryopsida). Ph.D. dissertation, Pennsylvania State University, University Park

Hedenäs, L. (2005). Bryophyte flora of Uganda. 4. Rhytidiaceae, Hylocomiaceae and Hypnaceae (Part 1). *Journal of bryology*, *27*(1), pp.55-66.

Huttunen, S., Bell, N. and Hedenäs, L. (2018). The Evolutionary Diversity of Mosses–Taxonomic Heterogeneity and its Ecological Drivers. *Critical reviews in plant sciences*, *37*(2-3), pp.128-174.

Jennings, O.E. (1913). A manual of the mosses of western Pennsylvania. The author, Press of the City Mission Pub. Co. doi:10.5962/bhl.title.54494

Klazenga N. (2012). Australian Mosses Online. 28. Orthorrhynchiaceae. http://www.anbg.gov.au/abrs/Mosses_online/Orthorrhynchiaceae.pdf

Klazenga, N. (2012) Australian Mosses Online. 29. Pterigynandraceae. http://www.anbg.gov.au/abrs/Mosses_online/Pterigynandraceae.pdf

Kruijer H. (2002). Hypoterygiaceae of the world. Blumea, Suppl. 13:1–388.

Milne J., Klazenga N. (2012) Australian Mosses Online. 24. Entodontaceae. http://www.anbg.gov.au/abrs/Mosses_Online/Entodontaceae.pdf

Ning-Ning Yu & Yu Jia (2012) The taxonomic status of two species of *Calyptothecium* Mitt. (Pterobryaceae, Bryopsida), Journal of Bryology, 34:1, 63-65. doi: 10.1179/1743282011Y.0000000044

Paton, J.A., Pearce, J.V. (1957). The Occurrence, Structure and Functions of the Stomata in British Bryophytes. Transactions of the British Bryological Society 3, 228–259. doi:10.1179/006813857804829560

Pursell, R. A. (1987). A taxonomic revision of *Fissidens* subgenus *Octodiceras* (Fissidentaceae). Mem. New York Bot. Gard. 45: 639-660.

Pursell, R. A., Bruggeman-Nannenga, M. A. Allen, B. H. (1988). A taxonomic revision of *Fissidens* subgenus *Sarawakia* (Bryopsidae: Fissidentacaea).

Smith, A.J. (2004). The Moss Flora of Britain and Ireland. Cambridge, UK: Cambridge University Press.

Tangney, R.S. (1997). A Generic Revision of the Lembophyllaceae. J. Hattori Bot. Lab 81, 123–153.

Touw A. (1971). A taxonomic revision of the Hypnodendraceae (Musci). Blumea 19: 211-354.

Vitt, D.H., Buck, W.R. (1984). The Familial Placement of Bryowijkia (Musci: Trachypodaceae). Brittonia 36, 300. doi:10.2307/2806531

Welch, W.H., (1943). The Systematic Position of the Genera Wardia, Hydropogon, and Hydropogonella. The Bryologist 46, 25.

Yu, N.N., Jia, Y. (2012). The taxonomic status of two species of Calyptothecium Mitt. (Pterobryaceae, Bryopsida). Journal of Bryology 34, 63-65.

Zander, R.H., Eckel, P.M. (1993). Genera of the Pottiaceae: mosses of harsh environments. Buffalo, New York: Buffalo Society of Natural Sciences.
